# Variola Vaccinæ or Vaccinia, or Cow-Pock

**Published:** 1838-08-29

**Authors:** N. Chapman

**Affiliations:** Professor of the Theory and Practice of Physic, in the University of Pennsylvania


### THE MEDICAL EXAMINER.

DEVOTED TO MEDICINE, SURGERY, AND THE COLLATERAL SCIENCES.

EDITED BY J. B. BIDDLE, M. D. AND M. CLYMER, M. D.

No. 18.] PHILADELPHIA, WEDNESDAY, AUGUST 29, 1838. [Vol. I.

### VARIOLA VACCINÆ OR VACCINIA, OR COW-POCK.

*By* N. Chapman, M. D., *Professor of the Theory and Practice of Physic, in the University of Pennsylvania.*

(Continued from page 268.)

Cutaneous affections of any kind, existing to some extent, the operation is inadmissible. It is not easy in this state of the skin to get the virus to act, and when it does, it is apt (as I shall presently show) to generate a mixed disease devoid of the protective power. Even the sulphurous impregnation of the skin, which takes place in the cure of itch, we are told by Jenner, prevents the vaccine infection. To this purport, he relates the fact of his inability to communicate the disease to a body of soldiers in this condition, whose surface being cleansed by the warm bath, they very readily received it.

Much has been said concerning the selection of the virus. The foreign writers, including Jenner, seem generally to prefer the pellucid fluid directly from the vesicle, and urge the taking of it on or before the ninth day, or previously to its becoming opaque and purulent, or the areola being formed. That it is less active after this time, is well established. The use, however, of the fluid, has been, for many years, altogether abandoned in this city, and the scab substituted, to which we were led by the following motives:

1st. It allows the disease to run its course, free from the danger of changing its specific character by any artificial interference, or molestation.

2d. The virus embodied in the scab, by proper precautions, may be much longer preserved, even for a year or more, without vitiation or diminution of power.

3d. It supplies a larger amount of matter for an extensive propagation of the disease, and the difficulty of infection is lessened for the same reason.

Notwithstanding these recommendatory considerations, I am not satisfied that we have done right in adopting the scab. European practitioners of the most enlarged experience, have entered their protest against it. By all, it is admitted, that the opaque or purulent fluid is far less to be depended upon, than the pellucid lymph—and it is not to be conceived that the former, when dried into a scab, should acquire any new, or regain additional efficacy. It will, too, be hereafter seen, that on the prevalence of epidemic small-pox among us, the failures of vaccination, were infinitely more numerous than elsewhere—which may, with probability, be referred to this cause.

Be this as it may, no one doubts that there is a choice in the scabs—certain ones, only, being entitled to confidence. Those which are hard and compact, of a dark mahogany colour, and with a regular, well defined margin, should be selected. The pale grayish scab, scaly or lamellated in its structure, with ragged edges, is always suspicious, very liable to fail, or if it infects, produces an illegitimate disease, impotent to any security against small-pox.

Employing the scab, the loose, fuzzy parts which lie on the inner surface, and attach to the circumference, are to be scraped off, and a small portion of the real solid scab is to be powdered, and moistened, to the state of a thick or ropy fluid. As in the case of the pellucid lymph, this may be inserted into a small puncture or scratch, or what succeeds better, to lay it on the skin, and work it in with the point of a lancet, taking care not to penetrate so deep as to occasion bleeding, which is apt to defeat the operation, by diluting the virus to inertness, or more likely by washing it away.

The history of the progress of the disease, may be very succinctly told. It almost invariably happens, that the incision heals, so as scarcely to leave a vestige—any appearance to the contrary, denoting common inflammation, instead of the specific action of the virus. The infection succeeding, there may be usually seen on the close of the third, or the beginning of the fourth day, a small red speck, somewhat elevated, which on pressure imparts to the finger the sensation of its enclosing a grain of some hard substance. This minute pimple gradually enlarges—and about the sixth day, a small vesicle is formed out of it, having a round margin, flat surface, with a slight indentation in the centre—is of a pink colour, which changes to a deeper red, with a mixture of blue, and is darkest in the middle. There is, at the same time, thrown close around its base, a narrow efflorescence like a ring. On the eighth, ninth, or tenth day, for the period is not very precise, the vesicle is changed into a pustule, and the areola becomes more florid, and of half an inch, more or less, in diameter. The pustule soon after attains its height, and the efflorescence thoughout its whole extent is tumefied, in which state it continues for several days, then subsides and fades away. In the declination of the pustule, the centre darkens first, and the whole by degrees is converted into a hard smooth crust, of a mahogany complexion. The crust drops off spontaneously in the course of the third week, leaving a small superficial cellulated definite cicatrix.

This is the developement of the local affection. On the expiration of the seventh day, in children somewhat advanced, or adults, or those still older, in whom there is usually most disease, the ordinary symptoms of fever are manifested, and sometimes even to a considerable height.

Yet these are generally slight and evanescent in infancy, the only serious complaint being soreness and tumefaction under the axilla, which even in infants exist, and hence care should be taken that they be not raised up by the arms.

Connected with the history of vaccination, there are one or two other circumstances deserving of notice. The local affection is sometimes very early in its developement and rapid in its career, appearing on the second day, and reaching maturity in four or five days, though oftener the reverse, and especially as regards the slowness of its disclosure. I have known it to be postponed in one instance, to the fourteenth, in another to the twenty-first day—and there is a case recorded in which the period was extended to six weeks. But what is more extraordinary in this last case, a second vaccination took effect, and subsequently the first one became completely evolved. An instance, too, is mentioned where the operation failing in a child, it was repeated in ten days successfully, the disease going regularly through its several stages—and six months afterwards, the former one began to inflame, and finally presented a genuine pustule, leaving behind it a regular cicatrix.*

Nearly always, there is a solitary pustule produced by the act of vaccination, and that at the point of the insertion of the matter. But occasionally we meet with a few vesicles of an imperfect character around the areola of the original or parent pustule. Cases, also, though very rarely, have been noticed, of a sparse and scattered eruption on the body. Thus we learn from the report of the Central Vaccine Committee of France, that in 1818-19, there was a considerable number of instances, in which many pustules occurred, so completely formed that matter taken from them, produced the genuine disease.

This, then, is a brief account of the legitimate form of the vaccine affection, with its occasional anomalies.

More, perhaps, than any other point, is it important to understand the diagnosis between the genuine and the spurious disease, and no great attention is usually required to decide it with proper discrimination. It may, however, facilitate the comprehension of the distinctions between them, to bring the two affections into immediate contrast.

1st. There is, in the legitimate disease, no evidence, ordinarily, of successful infection till the third day—and then we are presented with a minute and elevated pimple, having a definite margin, and flattened surface. The spurious, on the contrary, shows itself very soon in the form of a phlegmon, with considerable inflammation.

2d. The pimple in the genuine disease, gradually increases till about the sixth day, when it is converted into a vesicle containing pellucid lymph, retaining the same figure and construction: whereas, in the spurious, with its original phlegmonous character, it reaches maturity before this period, and becomes an abscess filled with pus.

3d. In the genuine disease, the vesicle changes to a pustule from the ninth to the tenth day, at which time it is surrounded by a very regular areola. But long before this period, the abscess in the spurious has ruptured and scabbed, or degenerated into a ragged sore, and in place of a defined areola, has about it a diffusive erysipelatus blush.

4th. The pustule of the genuine disease, when at its height, is round, or oval, and elevated, with a perfectly definite margin, flattened surface, and a central depression, resembling a button mould bound tightly by the skin, to which it has indeed been compared. Directly the reverse are the figure and condition of the local affection in the spurious, which now, and from the beginning, looks like a common fester, or boil, being conical, or pointed.

5th. There is an equal difference in the cicatrix or scar, left behind. In the genuine it is small, striated, and cellulated. That of the spurious, on the contrary, being scarcely perceptible, or very large, smooth, and polished.

No difficulty of discrimination could well be experienced, were the two states of the disease always thus orderly in their course, and strongly characterized. But it occasionally happens, from a feebler developement, or some injury done to the vesicle or pustule, or by some other cause, its aspect and condition are so changed, as to embarrass exceedingly a decision regarding its nature and protective efficacy. Examples to this purport, are found especially in vesicles, or pustules of much smaller than the usual dimensions, of a paler or more pearly colour, or the want of an areola, or with a very slight one, or, on the contrary, exceedingly extensive and undefined, like an erysipelatous blush, or so rubbed or torn, as to be deprived of their distinctive signs. Cases of such ambiguity occurring, or, in short, where, for any reason, there is the slightest doubt of the success of the operation, it becomes our duty to repeat it without delay.

Not a word need be said of the prognosis. This is always favourable—no instance of death, so far as I know, having happened from the disease, or, indeed, of any danger attending it. The anatomical characters are hence confined to the pustule itself, and here I may refer to what was said in regard to that of small-pox, they being essentially the same in the two affections; so far, at least, that in each there is the peculiar cellulated structure, formerly described. I leave its pathology to be deduced from the facts which have been stated, and general analogy.

Of the treatment of the disease it may be observed, that children are seldom so sick as to demand medicine, or even any material change of diet. Evacuations of the bowels, by the mildest laxatives, and occasionally small doses of the dulcified spirit of nitre, or antimonial wine, I have found sufficient in the worst of their cases. But, should an attack assume a more violent shape, which now and then occurs in grown people, the management is to be conducted on ordinary principles, and by the customary means, suited to the particular indications.

The local affections, however, more frequently call for attention. To allay excess of inflammation, cold water, or water and vinegar, or the diluted acetate of ammonia, or a weak solution of acetate of lead, or a saturnine poultice, may be applied; and to arrest or heal the ulcer, the common dressings are to be used. In some of these latter instances, however, I have found the blue mercurial ointment, or the citron ointment, or simple cerate with calomel, singularly serviceable.

Nothing is required to prepare the system for the reception of the disease, or its subsequent purification, as is vulgarly believed, from the taint which it imbibes. This part of the subject, I shall conclude with advising that, after using all the precautions already directed, the patient is to be visited

* Journal of Foreign Medicine, vol. iii. p. 719.

on the appearance of the vesicle, at its maturity, in the pustular state,—and on its declination,—watching, to determine whether it goes through these several stages with regularity, and, finally, to examine the cicatrix.

The question may here be very properly asked, in consequence of recent and multiplied reports to the prejudice of vaccination, whether, on the whole, it is still entitled to confidence? There was no time, even in the season of its greatest triumph, that allegations were not made, of the occasional failure of the process. But, perhaps, in a fair estimate, such instances amount to little, and may be so explained, as not materially to affect the value of the practice.

The sources of miscarriage I shall endeavour to indicate, and which are thought mainly, though not altogether, to proceed from the use of impure matter.

1st. The udder of the cow is liable to two species of pustules bearing an analogy to each other—the one secreting genuine, and the other a spurious virus, having no preventive efficacy against small-pox. An ignorance of this fact, led to some failures in the early period of vaccination. The illegitimate disease in the cow, is characterized by nearly the same circumstances as in the human subject, both as to the local and constitutional affections.*

2d. As regards our own species, the vesicle may be originally spurious from several causes, among which the practice of deriving virus from an individual who had previously undergone the variolous or vaccine disease. It was a common opinion at an early period of vaccination, that the genuine vaccine pustule might be induced in a system thus circumstanced. The repetition of the process of vaccination, was indeed, as well as I recollect, recommended by Jenner himself, as the best means of transmitting the virus to distant countries. But the fallacy of the opinion has long since been exposed. As demonstrated by an infinity of trials, the vaccine and variolous matter has on such a system only the power of creating local inflammation, or, at most, a phlegmon, like that excited by other irritants or poisons. Once exposed to their specific operation, it loses, for a time at least, its susceptibility to their influence—or if instances to the contrary occur, they are to be deemed as mere exceptions, not affecting the general principle. Labouring under the error to which I have alluded, practitioners did much to spread an illegitimate disease.

3d. The vesicle may be spurious from its existing in a distempered subject. By Jenner, the power of certain cutaneous disorders over vaccination, was early detected, and has since been more particularly pointed out by some other writers. Bateman says, "the most frequent cause of the deterioration of the lymph, seems to be the presence of chronic cutaneous eruptions, or the concurrence of eruptive fevers, or even of other febrile diseases. The chronic cutaneous affections, which sometimes impede the formation of the genuine vaccine vesicle, have been described by Jenner, under the ordinary indefinite term, Herpes, and Tinea Capitis. But in the more accurate phraseology of Dr. Willan, they are herpes, (including the *shingles* and vesicular ringworm,) psoriasis, and impetigo, (the dry and humid tetter,) the lichen, and most frequently the several varieties of porrigo, comprising the contagious eruptions," as the itch especially. We are further told by Tierny, that ulcers, as well as recent wounds, are also mischievous in their tendencies.

Even slighter occurrences, in the opinion of Jenner, have an effect. Not long before his death,* in a circular letter addressed to the profession, he declares that "mere abrasions of the cuticle, such, for example, as are found in the nurseries of the opulent, as well as in the cottages of the poor, behind the ears, and on many other parts, where the cuticle is tender," are pernicious in this way. "We find," continues he, "irregularity in the vaccine vesicle, if the skin is beset with herpetic blotches, or even simple serous oozings from an abraded cuticle; a speck behind the ear, which might be covered with a split pea, is capable of disordering the vaccine vesicle."

This is really ultraism. Could it be established, that vaccination is controlled by such trivial occurrences, it would constitute a more serious objection to it than any which have been alleged by its most inveterate foes. The fact is, that Jenner latterly, pressed on all sides by the augmented and cumulative proofs of the fallibility of vaccination, lost his candour, and from a sort of parental partiality for his discovery, sought to vindicate it, or explain away its imperfections, in a manner unworthy of his former reputation for philosophical truth.

He, however, rendered it probable, by a series of observations, that the vaccine action will enter into combination with certain species of herpes, producing a third disease of a hybridous nature, which may be indefinitely propagated by inoculation, without change of character, though ineffectual to all the purposes of preservation against variola.

It was early observed, and which lends confirmation to this statement, that vaccination practised in situations simultaneously exposed to the variolous infection, the case received a very material modification. Thus, at the first introduction of vaccination, Woodville, who had charge of the large small-pox hospital in London, instituted some experiments in that establishment, with a view of testing the validity of Jenner's reports, and found, that in about three-fifths of the cases, a disease was produced, more or less of an eruptive nature, very different from the pure vaccine, and approaching more to variola. This led to a controversy between him and Jenner, which was soon settled by the discovery, that the anomaly could be directly traced to the operation of the variolous effluvia with which the atmosphere of the wards of the hospital was impregnated, where the patients were placed, though not till great mischief had been done by the dissemination of lymph derived from this polluted source.†

Of the influence of the other eruptive fevers, we are not so accurately informed. It seems, however, that when measles, or scarlatina, breaks out, the vaccine vesicle is arrested, till these fevers abate, when it again resumes, and finishes its

* Good, 395, vol. ii.

* 1821. † Marshall on Vaccination.

progress, with a retention of all its peculiar properties.*

3d. Matter originally pure, may, by keeping, undergo some change, weakening or destroying its qualities. Time, which alone will cause such effects, is much aided by a high degree of temperature. During winter, the pellucid virus, which is more perishable than the scab, may be preserved for many months: whereas, in summer, it loses its strength in a few days, and in some instances, even in a few hours. Being only impaired, it causes a pustule so imitative of the genuine one, as hardly to be discriminated. The particular, indeed, in which they chiefly differ, is, that in the former, the scab is said prematurely to fall off, leaving the constitution so slightly affected, that no protection is afforded.

An account is given by Baron Humboldt, of a surgeon of Lima, having vaccinated a number of persons with superannuated matter, brought to that city, all of whom apparently did well, though the whole subsequently received variola by inoculation, in a very mild shape. To the same point, cases might be cited, from various records, showing that virus enfeebled by age or otherwise, will excite a simulated affection, which, while it is incapable of an entire resistance, tempers the violence of small-pox.

4th. An effect, not altogether dissimilar, is said to follow the use of virus from an immature vesicle. Thus we are informed by Willan, that if lymph be taken from a vesicle too early, it often proves totally inefficient, and where it does operate, the genuine disease is not produced.

5th. Matter may become degenerate in a vesicle originally genuine. This often happens from the subversion of the specific action of the vesicle by lacerating it to get the virus, or from its being accidentally rubbed, or otherwise molested in its progression.

6th. The genuine pustule may be local, extending no security whatever to the system at large, and thus constituting another source of failure. Not the least striking fact of this nature, came within my own observation, so early as the year 1804. By the late Dr. Stewart, then physician to the dispensary of this city, a man was vaccinated, who seemingly having the genuine disease, matter was taken from his pustule, with which several of his children were successfully infected. The father, after a while, broke out with the natural small-pox, and had it severely—the children, however, escaped, and resisted repeated variolations, as well as vaccinations. In the fifth volume of the Medical and Physical Journal, a case precisely similar, is related by Dr. Harrison, and I have no doubt that others are to be met with.

Lastly. There is reason to suspect, that certain states of the atmosphere, or other occult physical causes, have an influence over the process. Gregory has correctly remarked, that it occasionally happens, that many spurious cases of the disease appear at the same time, and more at the approach of winter, than either in the spring or summer months. The same fact I have observed myself, though, I think, oftener in very hot weather, and have been in the habit of referring it to the well known operation of heat in the deterioration of the virus. Certainly, it is less efficient under such circumstances, as shown by the greater difficulty of imparting the disease by inoculation.

To establish a test of the efficiency of vaccination, has engaged much attention, and various modes have been suggested, among which revaccination some three or four days after the first operation, was very confidently proposed. It is said, that if the first vaccination be perfect, or in other words, the constitution is adequately affected by it, the vesicle of the second will be so accelerated, that the areola around each takes place simultaneously, both moving on, *pari passu*, and fade together.* But I presume, this proposition involves some fallacy, as it seems not to have been generally adopted, and of late, we hear nothing of the practice. To revaccinate at some period after the case is over, is more common. The system being protected, it loses its susceptibility to the vaccine impression, and instead of a genuine vesicle, a slight erysipelatous inflammation, or small phlegmon, ensues, which usually soon subsides. But in the latter particular it may be otherwise, and I have seen very sore arms, painful axillary swellings, and fever, thus induced. Nor can the expedient be entirely trusted. Either from defect of the virus, or peculiar condition of the system at the time, the second operation may be defeated, or run the course I have described. It affords, at best, only negative proof. Yet, on the whole, it is to be preferred. The objection to variolation as a test, most strongly urged, is, that we must keep up small-pox, to supply matter for the purpose, and that it is liable to the same fallacies as revaccination. The scar so greatly relied on, though it may show that a genuine pustule has existed, affords no evidence of the general disease, or that the system at large is duly protected. That which is most to be regarded, "is distinct, circular, radiated, and cellulated, and above all is so small, that it may be covered with a pea." This is the language of Gregory, who from his ample experience as physician to one of the large vaccine institutions of London, is well entitled to be heard on the subject.

It is now the practice of the vaccinists of Great Britain, more particularly, to proceed on the supposition, that security is best attained by the multiplication of cotemporaneous vesicles.

"As a general rule," says Mr. Moore,† "it may be advisable to make two punctures in each arm, and when this is properly done, three vesicles, at least, will commonly arise, and if four are excited, it is never to be regretted. If only two vesicles arise, neither should be opened or disturbed,—and if the vaccine proceeds regularly to the end, the vaccination may be considered complete. When three or more vesicles have been excited, lymph may be taken from the subject. But it is prudent always to leave two complete vesicles to pass through their course untouched." ‡

* Gregory has recorded an instance where vaccination was retarded for sixteen days, during which time measles had possession of the system. Genuine varicella, on the contrary, he says, does not at all interfere with it, the two affections running their courses harmoniously—which, however, is disputable.

* Bryce on Vaccination.

† History of the Practice of Vaccination.

‡ These are the directions also of the London Vaccine Establishment.

Though emanating from such high authority, I confess that I do not approve of this practice. Even allowing that the multiplication of punctures increases the chances of infection, it cannot tend to ensure the production of the genuine disease. It seems to me, that the suggestion originated in pathological views altogether false, and is without the sanction of any adequate experience of its utility. The system loses its susceptibility to small-pox, not by the quantity of vaccine virus introduced, but by the impression it creates, and to do which, provided it acts, one particle is as effectual as ten thousand. To believe that the living body is capable of saturation by an excess of matter, as happens in a chemical process, which seems to me to be the foundation of this creed, is a conclusion drawn from a very remote analogy, and in itself is futile and absurd. By Thompson, whose inquiries have been so accurately conducted on every point connected with vaccination, it is also stated, that the failures have been as numerous where three as one pustule was raised.* It is, indeed, not a little extraordinary, that such an expedient should have been adopted, after the decided experiments of Kamper, formerly referred to, in relation to small-pox, which so conclusively showed that a single inoculation proved as effective to all intents and purposes as seven, the number which he tried. As a criterion of constitutional affection in this case, I am apprehensive that we are destitute of any, deserving of entire confidence, and that such an attainment is still ardently to be desiderated.

Notwithstanding this, and perhaps some other objections, it follows, I think, on the whole, from what has been said, that the common allegations against vaccination in relation to the ancient form of small-pox, are not well sustained, proceeding rather from the carelessness of the practitioner, than the demerits or imperfections of the expedient. We have, in proof of this, the strong fact, that prior to the occurrence of the varioloid epidemic, no instance of failure occurred in the practice of Jenner, or his nephew Mr. Jenner, and not above eight or ten in that of the National Vaccine Establishment of London, where vaccination was done to an enormous extent. Confirmatory of the same conclusion, we learn from an official report to the British government in 1812, apparently drawn up with care, that out of 2,671,662 cases of vaccination, only seven cases of small-pox had occurred. Can any other reason be required to induce the public to confide, exclusively, the practice to the skilful and experienced?

Of the alleged failures, some, and perhaps many, were not really so. It is admitted, that varicella may be sometimes confounded with small-pox. By Willan, whom, I have said, was, above all men, skilled in the diagnosis of this order of diseases, it is declared, that in six years he saw seventy-four cases of chicken-pox, which had been mistaken for small-pox after vaccination.

Conceding, however, to the fullest extent claimed by its opponents, the failure of vaccination, the instances are probably not more numerous than of variolation. But this, and all else, which I have said, apply only to a former state of things. We are henceforward to contemplate the subject in a far less favourable point of view—and here a stage in the inquiry is reached, when it becomes proper to introduce some account of the varioloid epidemic.

* Thompson on Varioloid disease, p. 314.

(To be continued.)

